# Supplementary material for: High-Fat Diet with Normal Caloric Intake Elevates TMA and TMAO Production and Reduces Microbial Diversity in Rats
Source: Nutrients. 2025 Jul 5;17(13):2230. doi: 10.3390/nu17132230 (PMC12252406; doi:10.3390/nu17132230)
Supplement: Supplementary file 1 [file nutrients-17-02230-s001.zip › nutrients-3700168-supplementary.pdf]

C 1090 - 10

control diet with w/10% energy from fat

Metabolized energy

Content	Value		unit
Fat	358	(10%)	kcal/kg
Protein	828	(24%)	kcal/kg
Carbonhydrates	2,329	(66%)	kcal/kg

crude nutrients and moisture

Content	Value		unit
Moisture	79,090	(7.9%)	mg/kg
Crude Ash	43,274	(4.3%)	mg/kg
Crude Fibre	30,829	(3.1%)	mg/kg
Crude Fat	39,725	(4.0%)	mg/kg
Crude Protein	206,900	(20.7%)	mg/kg
Nitrogenfree extractives	600,183	(60%)	mg/kg

Carbonhydrates

Content	Value	unit
Monosaccharides	15,143	mg/kg
Disaccharides	117,705	mg/kg
Polysaccharides	427,227	mg/kg

Minerals

Content	Value	unit
Calcium	7,402	mg/kg
Potassium	5,703	mg/kg
Magnesium	558	mg/kg
Sodium	1,890	mg/kg
Phosphorus	6,522	mg/kg

Altromin Spezialfutter GmbH & Co. KG
Im Seelenkamp 20, 32791 Lage, Germany
info@altromin.de - www.altromin.com

Trace elements

Content	Value	unit
Aluminium	3.61	mg/kg
Chlorine	2,992.50	mg/kg
Iron	134.70	mg/kg
Flourine	3.13	mg/kg
Iodine	0.41	mg/kg
Cobalt	0.12	mg/kg
Copper	4.46	mg/kg
Manganese	75.75	mg/kg
Molybdenum	0.15	mg/kg
Sulfur	2,779.54	mg/kg
Selenium	0.29	mg/kg
Zinc	24.16	mg/kg

Added vitamins

Content	Value	unit
Vitamin A	15,000	IU/kg
Vitamin D3	500	IU/kg
Vitamin E	150	mg/kg
Vitamin K3	10	mg/kg
Vitamin B1	20	mg/kg
Vitamin B2	20	mg/kg
Vitamin B6	15	mg/kg
Vitamin B12	43	µg/kg
Nicotinic acid	50	mg/kg
Pantothenic acid	50	mg/kg
Folic acid	10	mg/kg
Biotin	201	µg/kg
Choline chloride	1,013	mg/kg
Vitamin C	20	mg/kg

Altromin Spezialfutter GmbH & Co. KG
Im Seelenkamp 20, 32791 Lage, Germany
info@altromin.de - www.altromin.com

Amino acids

Content	Value	unit
Alanine	3,134	mg/kg
Arginine	11,620	mg/kg
Aspartic acid	4,774	mg/kg
Cystine	3,747	mg/kg
Glutaminc acid	28,926	mg/kg
Glycine	4,141	mg/kg
Histidine	6,283	mg/kg
Isoleucine	8,898	mg/kg
Leucine	17,033	mg/kg
Lysine	20,604	mg/kg
Methionine	8,609	mg/kg
Phenylalanine	8,654	mg/kg
Proline	15,389	mg/kg
Serine	6,481	mg/kg
Threonine	8,608	mg/kg
Tryptophan	2,404	mg/kg
Tyrosine	11,073	mg/kg
Valine	4,320	mg/kg

Fatty acid

Content	Value	unit
Arachidic acid C-20:0	340	mg/kg
Eicosanoic acid C-20:1	153	mg/kg
Alpha-Linolenic acid C-18:3	333	mg/kg
Linolenic acid C-18:2	2,059	mg/kg
Palmitic acid C-16:0	5,240	mg/kg
Stearic acid C-18:0	3,793	mg/kg
Oleic acid C-18:1	11,300	mg/kg

Altromin Spezialfutter GmbH & Co. KG
Im Seelenkamp 20, 32791 Lage, Germany
info@altromin.de - www.altromin.com

C 1010

carbohydrate rich diet

Metabolized energy

Content	Value		unit
Fat	454	(12%)	kcal/kg
Protein	683	(18%)	kcal/kg
Carbonhydrates	2,635	(70%)	kcal/kg

crude nutrients and moisture

Content	Value		unit
Moisture	49,870	(5.0%)	mg/kg
Crude Ash	41,708	(4.2%)	mg/kg
Crude Fibre	15,170	(1.5%)	mg/kg
Crude Fat	50,450	(5.0%)	mg/kg
Crude Protein	170,750	(17.1%)	mg/kg
Nitrogenfree extractives	672,052	(67.2%)	mg/kg

Carbonhydrates

Content	Value	unit
Monosaccharides	66,500	mg/kg
Disaccharides	441,105	mg/kg
Polysaccharides	133,527	mg/kg

Minerals

Content	Value	unit
Calcium	6,193	mg/kg
Potassium	4,736	mg/kg
Magnesium	480	mg/kg
Sodium	1,665	mg/kg
Phosphorus	5,557	mg/kg

Altromin Spezialfutter GmbH & Co. KG
Im Seelenkamp 20, 32791 Lage, Germany
info@altromin.de - www.altromin.com

Trace elements

Content	Value	unit
Aluminium	2.41	mg/kg
Chlorine	2,420.00	mg/kg
Iron	119.26	mg/kg
Flourine	2.78	mg/kg
Iodine	0.36	mg/kg
Cobalt	0.09	mg/kg
Copper	3.88	mg/kg
Manganese	67.24	mg/kg
Molybdenum	0.13	mg/kg
Sulfur	2,484.52	mg/kg
Selenium	0.25	mg/kg
Zinc	21.29	mg/kg

Added vitamins

Content	Value	unit
Vitamin A	15,000	IU/kg
Vitamin D3	500	IU/kg
Vitamin E	180	mg/kg
Vitamin K3	10	mg/kg
Vitamin B1	20	mg/kg
Vitamin B2	20	mg/kg
Vitamin B6	15	mg/kg
Vitamin B12	41	µg/kg
Nicotinic acid	50	mg/kg
Pantothenic acid	50	mg/kg
Folic acid	10	mg/kg
Biotin	201	µg/kg
Choline chloride	1,012	mg/kg
Vitamin C	20	mg/kg

Altromin Spezialfutter GmbH & Co. KG
Im Seelenkamp 20, 32791 Lage, Germany
info@altromin.de - www.altromin.com

Amino acids

Content	Value	unit
Alanine	2,376	mg/kg
Arginine	9,736	mg/kg
Aspartic acid	3,455	mg/kg
Cystine	3,156	mg/kg
Glutaminc acid	23,314	mg/kg
Glycine	3,060	mg/kg
Histidine	5,221	mg/kg
Isoleucine	7,149	mg/kg
Leucine	14,531	mg/kg
Lysine	17,344	mg/kg
Methionine	7,185	mg/kg
Phenylalanine	7,077	mg/kg
Proline	12,586	mg/kg
Serine	5,169	mg/kg
Threonine	7,082	mg/kg
Tryptophan	1,965	mg/kg
Tyrosine	9,203	mg/kg
Valine	3,206	mg/kg

Fatty acid

Content	Value	unit
Arachidic acid C-20:0	50	mg/kg
Eicosanoic acid C-20:1	150	mg/kg
Alpha-Linolenic acid C-18:3	150	mg/kg
Linolenic acid C-18:2	28,500	mg/kg
Palmitic acid C-16:0	2,500	mg/kg
Stearic acid C-18:0	1,350	mg/kg
Oleic acid C-18:1	13,500	mg/kg

Altromin Spezialfutter GmbH & Co. KG
Im Seelenkamp 20, 32791 Lage, Germany
info@altromin.de - www.altromin.com

C 1090 - 45

obesity-inducing diet with w/45% energy from fat (22%fat)

Metabolized energy

Content	Value		unit
Fat	2,037	(45%)	kcal/kg
Protein	831	(18%)	kcal/kg
Carbonhydrates	1,629	(37%)	kcal/kg

crude nutrients and moisture

Content	Value		unit
Moisture	39,075	(3.9%)	mg/kg
Crude Ash	39,100	(3.9%)	mg/kg
Crude Fibre	55,707	(5.6%)	mg/kg
Crude Fat	226,300	(22.6%)	mg/kg
Crude Protein	207,775	(20.8%)	mg/kg
Nitrogenfree extractives	432,043	(43.2%)	mg/kg

Carbonhydrates

Content	Value	unit
Monosaccharides	102,200	mg/kg
Disaccharides	50,355	mg/kg
Polysaccharides	229,252	mg/kg

Minerals

Content	Value	unit
Calcium	7,988	mg/kg
Potassium	7,155	mg/kg
Magnesium	651	mg/kg
Sodium	2,363	mg/kg
Phosphorus	5,882	mg/kg

Altromin Spezialfutter GmbH & Co. KG
Im Seelenkamp 20, 32791 Lage, Germany
info@altromin.de - www.altromin.com

Trace elements

Content	Value	unit
Aluminium	2.97	mg/kg
Chlorine	4,220.00	mg/kg
Iron	119.31	mg/kg
Flourine	3.33	mg/kg
Iodine	0.31	mg/kg
Cobalt	0.10	mg/kg
Copper	3.78	mg/kg
Manganese	67.36	mg/kg
Molybdenum	0.19	mg/kg
Sulfur	1,522.08	mg/kg
Selenium	0.19	mg/kg
Zinc	17.43	mg/kg

Added vitamins

Content	Value	unit
Vitamin A	15,000	IU/kg
Vitamin D3	500	IU/kg
Vitamin E	150	mg/kg
Vitamin K3	10	mg/kg
Vitamin B1	20	mg/kg
Vitamin B2	20	mg/kg
Vitamin B6	15	mg/kg
Vitamin B12	32	µg/kg
Nicotinic acid	50	mg/kg
Pantothenic acid	50	mg/kg
Folic acid	10	mg/kg
Biotin	200	µg/kg
Choline chloride	1,002	mg/kg
Vitamin C	20	mg/kg

Altromin Spezialfutter GmbH & Co. KG
Im Seelenkamp 20, 32791 Lage, Germany
info@altromin.de - www.altromin.com

Amino acids

Content	Value	unit
Alanine	10,977	mg/kg
Arginine	12,804	mg/kg
Aspartic acid	11,759	mg/kg
Cystine	1,465	mg/kg
Glutaminc acid	29,145	mg/kg
Glycine	22,517	mg/kg
Histidine	3,470	mg/kg
Isoleucine	7,154	mg/kg
Leucine	7,553	mg/kg
Lysine	11,782	mg/kg
Methionine	4,416	mg/kg
Phenylalanine	6,978	mg/kg
Proline	19,684	mg/kg
Serine	7,906	mg/kg
Threonine	6,147	mg/kg
Tryptophan	1,300	mg/kg
Tyrosine	5,289	mg/kg
Valine	7,297	mg/kg

Fatty acid

Content	Value	unit
Arachidic acid C-20:0	0	mg/kg
Eicosanoic acid C-20:1	0	mg/kg
Alpha-Linolenic acid C-18:3	1,582	mg/kg
Linolenic acid C-18:2	2,260	mg/kg
Palmitic acid C-16:0	5,017	mg/kg
Stearic acid C-18:0	13,786	mg/kg
Oleic acid C-18:1	38,872	mg/kg

Altromin Spezialfutter GmbH & Co. KG
Im Seelenkamp 20, 32791 Lage, Germany
info@altromin.de - www.altromin.com

Supplementary Table S1.

	Average total energy intake /rat/experiment (kcal)	Average total food intake/rat/experiment (g)
Control diet	4560.06	1297.68
High-disaccharide diet	4520.90	1198.54
High-fat diet	4368.76	971.48

Supplemantary Table S2.

Initial body weight (g)	185.75 ± 7.34	182.26 ± 6.29	182.30 ± 5.69	P=0.83
Final body weight (g)	391.07 ± 26.07	384.59 ± 25.62	368.40 ± 23.02	P=0.67
